# Factors Associated with Adults’ Decisions to Undergo Rhinoplasty in Saudi Arabia: A Cross-Sectional Study

**DOI:** 10.3390/healthcare14203378

**Published:** 2026-10-10

**Authors:** Rasha Doumi, Abdulrahman Alhumaizi, Reem Abdullah Alfulayj, Lujain Rafa Alamri, Haifa Ali Alamro, Rahaf Saleh Alshahrani, Kadi Meshal Alotaibi, Razan Saad Alzhrani, Amel Fayed

**Affiliations:** 1 Department of Family and Community Medicine, College of Medicine, Princess Nourah Bint Abdulrahman University, Riyadh 11671, Saudi Arabia; aafayed@pnu.edu.sa; 2 Head and Neck Surgery Department, King Abdullah Bin Abdulaziz University Hospital, Riyadh 13412, Saudi Arabia; aahumaizi@hotmail.com; 3 College of Medicine, Princess Nourah Bint Abdulrahman University, Riyadh 11671, Saudi Arabia; reemiif6@gmail.com (R.A.A.); lujainalamri3@gmail.com (L.R.A.); alamro.haifaa@gmail.com (H.A.A.); srahaf747@gmail.com (R.S.A.); kadimeshaxx@gmail.com (K.M.A.); alzarazan4@gmail.com (R.S.A.)

**Keywords:** acceptance, rhinoplasty, complications, nose-related appearance concerns, Saudi Arabia

## Abstract

Background and objective: The growing global popularity of rhinoplasty underscores the need to better understand public opinions and choices about the procedure. This study examines how acceptance of cosmetic surgery, concerns about nose appearance, and peer influence are associated with the decision to consider rhinoplasty among Saudi adults. Methods: This cross-sectional study of 1591 participants used a self-administered online questionnaire. Data on considering or undergoing rhinoplasty, sociodemographic characteristics, and whether friends or family members had undergone the surgery were collected. The Acceptance of Cosmetic Surgery Scale (ACSS) and nose-related appearance concern scale were used. Perceived reasons for and against considering rhinoplasty, as well as perceived seriousness of postoperative complications, were examined. Data were analyzed using IBM SPSS v27. Results: The mean age was 35.7 ± 13.2 years, with a majority being females (70%). Of the 1591 participants, 74% were not considering rhinoplasty, while 26% were considering the procedure or had already undergone it. The most common perceived reason for undergoing rhinoplasty was to enhance appearance, while religious concerns were the most common perceived reason for refusing it. Participants identified the mismatch between the nose and the face (20.1%) as the most serious complication. The ACSS was a key factor associated with higher odds of considering rhinoplasty at all stages, from exploring options to undergoing surgery. During the exploration stage, those with financial constraints were more likely to explore rhinoplasty options, whereas wealthier participants were 67% less likely to do so (OR = 0.33, 95% CI: 0.16–0.71). Greater nose concerns and having a peer who had undergone surgery increased the odds of exploration and preparation. Older age was associated with higher odds of not considering rhinoplasty (OR = 1.11, 95% CI: 1.03–1.19). Being female was linked to higher odds of undergoing rhinoplasty (OR = 7.76, 95% CI: 1.73–34.75). Conclusions: More than a quarter of the sample was willing to or had already undergone rhinoplasty. Factors associated with higher odds of considering rhinoplasty included female gender, young age, limited financial resources, having a friend or family member who had the surgery, concerns about nose appearance, and the acceptance of cosmetic surgery in general.

## 1. Introduction

Cosmetic surgeries have increased globally in recent decades, reflecting evolving social perceptions of physical appearance, body image, and aesthetic enhancement [1]. Advances in surgical techniques, improved safety profiles, and the widespread influence of digital media have contributed to their growing acceptance across diverse populations [2]. Global statistics indicate that aesthetic procedures continue to increase annually, with more than 34.9 million cosmetic procedures performed worldwide in 2023 [3], reflecting the growing role of cosmetic interventions in modern health and social contexts. Social media exposure, celebrity culture, and evolving beauty standards have further increased societal consideration of aesthetic procedures [4]. Rhinoplasty is one of the most frequently performed facial cosmetic surgeries worldwide, and its frequency has been rising recently [3,5]. It serves both functional and aesthetic purposes. Its popularity has increased widely in Middle Eastern and Gulf countries, including Saudi Arabia, where it accounted for 59% of all plastic surgeries in 2019 [6]. Recent regional studies have reported considerable public interest in these surgeries [7,8].

The decision to undergo rhinoplasty is complex, often driven by both medical and psychological factors. While a significant number of patients cite purely aesthetic (23.3%) or purely functional (25.5%) reasons, over half (51%) seek to address both simultaneously [9]. This suggests that appearance and breathing function are deeply intertwined for many individuals. The psychological dimension is further highlighted by the fact that the top reason for revision surgery (50.16%) is not to correct a poor outcome but to pursue further aesthetic refinement [10]. This continual pursuit of enhancement may be rooted in pre-existing body image issues, as potential patients often report body dissatisfaction and low self-esteem [11]. Social media and societal norms further influence this decision by promoting idealized beauty standards and increasing peer acceptance [4,6,12]. Peer influence and exposure to friends or relatives who have undergone rhinoplasty significantly increase acceptance and intention through normalization and shared experiences [7]. Additionally, awareness of complications and satisfaction with prior outcomes can either discourage or reinforce the decision, depending on individual perceptions of risk and benefit [10,13]. Sociodemographic characteristics such as age, gender, educational level, and income have been shown to shape acceptance and willingness to undergo rhinoplasty [7].

Despite its popularity, rhinoplasty remains a complex surgical procedure with a significant and often underestimated risk of complications. Data indicate that nasal obstruction (37.91%) and sleep disturbances (37.7%) are the most frequently reported issues, followed by frequent colds (29.2%). Although less common, visible scarring still affects 6% of patients [12]. Evidence suggests that public awareness of rhinoplasty complications varies considerably across populations. Several local studies in Saudi Arabia reflect a perception gap [7,12], showing that although many individuals recognize common postoperative outcomes, overall knowledge of potential surgical complications remains limited, and even many participants believed the surgery was safe [13]. This suggests public unawareness of potential issues.

Research on rhinoplasty acceptance in Saudi Arabia presents a paradox, with studies indicating it is socially accepted by many, especially younger generations, while some groups hold conservative views about undergoing the procedure [14,15]. Public perception of rhinoplasty and its acceptance are not always clear and do not fully explain the intention to undergo surgery. Psychological determinants also play a central role in shaping attitudes toward cosmetic surgery, with self-esteem widely recognized as a key factor influencing body image perception and appearance-related behaviors. Many studies from Saudi Arabia have focused on rhinoplasty acceptance, awareness of complications, and satisfaction after surgery [6,7,8,9]. The decision to undergo rhinoplasty remains underexplored among Saudi adults. In the current study, we plan to investigate how the decision to consider rhinoplasty is related to acceptance of cosmetic surgery, concerns about nose appearance, and peer influence among Saudi adults.

## 2. Materials and Methods

This is an analytic, cross-sectional, nationwide survey conducted among adults aged 18 years or older residing in all regions of Saudi Arabia, with no gender restrictions. The data were collected using an online, self-administered questionnaire covering various concepts:

- Sociodemographic data: age, gender, marital status, monthly income, and highest level of education attained.
- The Acceptance of Cosmetic Surgery Scale (ACSS) was used to estimate acceptance of aesthetic surgery in general [16]. The 15-item ACSS, measuring attitudes toward cosmetic surgery across three dimensions (Intrapersonal, Social, and Consider), was utilized:
  ○ Intrapersonal: Measures self-oriented motivations, such as the desire for improved self-image or personal satisfaction.
  ○ Social: Assesses social motives, such as wanting to be more attractive to a partner or advance one’s career.
  ○ Consider: Evaluates the likelihood of considering cosmetic surgery based on practical factors, such as complications or pain.

Scoring of ACSS: Participants rate each of the 15 items on a 7-point Likert scale from “strongly disagree” to “strongly agree”. The total score is calculated by averaging the three subscale scores. Higher total scores indicate greater acceptance of cosmetic surgery. Internal consistency of the ACSS was assessed using Cronbach’s alpha, which demonstrated excellent reliability (0.95).

- Nose-related appearance concern scale: Eleven items were selected from The Derriford Appearance Scale (DAS59; Musketeer Press, Bradford on Avon, UK) [17] based on their relevance to the study objectives, with adaptation to focus on nose appearance. Response categories used a five-point Likert format (1 = strongly agree, 2 = agree, 3 = neutral, 4 = disagree, 5 = strongly disagree). The total score was 55 (range: 5–55), with lower scores indicating greater nose-related concerns. The internal consistency, as measured by Cronbach’s alpha, was excellent (0.97). Preliminary structural evaluation was conducted using principal component analysis for extraction. The correlation matrix between items ranged from 0.69 to 0.85, indicating that the items share an underlying factor. The Kaiser–Meyer–Olkin was 0.96, and Bartlett’s test of sphericity was significant (χ^2^ = 23,709, *p* < 0.05). The analysis identified one distinct factor that accounts for 79.8% of the total variance. This initial psychometric evaluation demonstrated structural unidimensionality and high internal consistency. (Detailed results are available in the Supplementary files.)
- Participants were asked to specify their stage in considering rhinoplasty, with the following options: “already have done the surgery,” “preparing for the surgery (had consultation/booked the surgery),” “just exploring options,” “not considering it at all,” and “did not think about it.” These five categories establish a straightforward, logical framework to assess their readiness, progressing from unawareness to action. This classification streamlines participant grouping and encompasses the full spectrum from rejection to completion.
- Participants’ perceptions of rhinoplasty: This study examined perceptions of rhinoplasty, including why people choose the procedure, why they might avoid it, and how seriously they perceive potential complications. Possible reasons for and against conducting the surgery were listed, and participants rated each reason on a 5-point Likert scale (strongly agree to strongly disagree). For analysis, we aggregated the “strongly agree” and “agree” responses and displayed them for each reason. Participants were asked to rate the severity of rhinoplasty complications on a Likert scale from 1 to 10 (1 = least severe, 10 = most severe). For analysis, we combined the severity scores of 7 or more into a single category and listed the complications in order of increasing severity as perceived by participants.

The face and content validity of the tools used were confirmed through an expert panel review, which verified the relevance and comprehensiveness of the items. The panel included otolaryngologists, public health specialists, and biostatisticians. The primary questionnaire was initially developed in English and then translated into Arabic using a forward–backward translation method to identify language errors, verify clarity, and ensure semantic equivalence. Its linguistic and cultural appropriateness for Saudis was then evaluated by the research team’s Saudi members and confirmed through a pilot study.

The minimum sample size was estimated using G*Power (version 3.1) for a chi-square goodness-of-fit test with the following parameters: a small effect size (W = 0.10), expected intention to undergo rhinoplasty of 47% [10], significance level (a) of 0.05, statistical power of 80%, and degrees of freedom = 4 (five outcome groups). This yielded a minimum of 1194 participants. After accounting for a 15% anticipated incomplete response rate, the number was 1404.

We recruited 1591 participants who met the inclusion criteria via convenience sampling between October and March 2025. The survey link was sent to potential participants via social networks such as X, WhatsApp, Telegram, and Instagram. We used a snowballing technique, encouraging participants to share the survey link on their social media networks to ensure widespread coverage. Despite using non-probability sampling techniques that might have introduced selective bias or underrepresentation of some groups, the large sample size enabled us to recruit a large number of participants with various experiences with rhinoplasty (those who have already undergone the surgery, those planning to undergo the surgery, and those not intending to undergo the surgery). Additionally, to ensure data quality, we used validated data tools and limited participation to one response per device. Incomplete responses were automatically deleted from the database before conducting the analysis (*n* = 89).

All statistical analyses were conducted using IBM SPSS Statistics for Windows, version 27 (IBM Corp., Armonk, NY, USA). Descriptive statistics, including frequencies and percentages, were used to summarize the sample characteristics. The chi-square test was used to assess associations between categorical variables, and Fisher’s exact test was used if the assumptions of the chi-square test were not met. The Kruskal–Wallis H test was used to compare scores on the ACSS and the nose-related appearance concern scales across groups.

A multinomial logistic regression model was used to identify independent factors associated with consideration of rhinoplasty. The outcome variable was the rhinoplasty consideration status (five categories), with “not considering rhinoplasty at all” as the reference category. We included factors that showed statistically significant association in the bivariate analysis, including participants’ age, gender, economic level, marital status, having a friend or family member who had undergone rhinoplasty, ACSS, and nose-related appearance concern scores, as independent factors. However, the ACSS “Consider” subscale directly assesses willingness to consider cosmetic surgery and therefore substantially overlaps with an outcome defined by considering rhinoplasty; including this subscale as a predictor may produce a partly circular association. So, in the regression analysis, ACSS without the “Consider” subscale was used as a predictor. The Odds Ratio (OR) and its 95% Confidence Interval (C.I.) were reported, and a *p*-value of less than 0.05 was considered statistically significant for all tests.

## 3. Results

The study included 1591 participants, with the majority being females (70%). The average age was 35.7 ± 13.2 years, with 51% aged 20–40 years and married (60%). Approximately 65% of the participants had sufficient income, and 66% had a university education or higher. Social media was the primary source of information about rhinoplasty, accounting for around 74% of participants. Nearly half of the participants (49%) had at least one relative or friend who had undergone rhinoplasty. Regarding participants’ current stage of consideration for rhinoplasty, 74% are not considering it at all, while 26% are considering the procedure, are planning for the surgery, or have already undergone it (Table 1).

Younger participants were more likely to consider and explore rhinoplasty options than older participants. Undergoing surgery was more common among females than males (2.3% versus 0.4%). Unmarried individuals were more likely to consider rhinoplasty. Participants with limited financial resources were significantly more open to considering and undergoing rhinoplasty than those with sufficient financial resources. There was a notable difference between individuals with friends or family members who already had rhinoplasty and those without. Among those with peers who had the procedure, nearly 30% were exploring or planning to have it, and about 3% had already undergone it, whereas only 17% of individuals without friends or family who had rhinoplasty were considering the procedure, and less than 1% had undergone it (Table 2).

**Figure 1:**
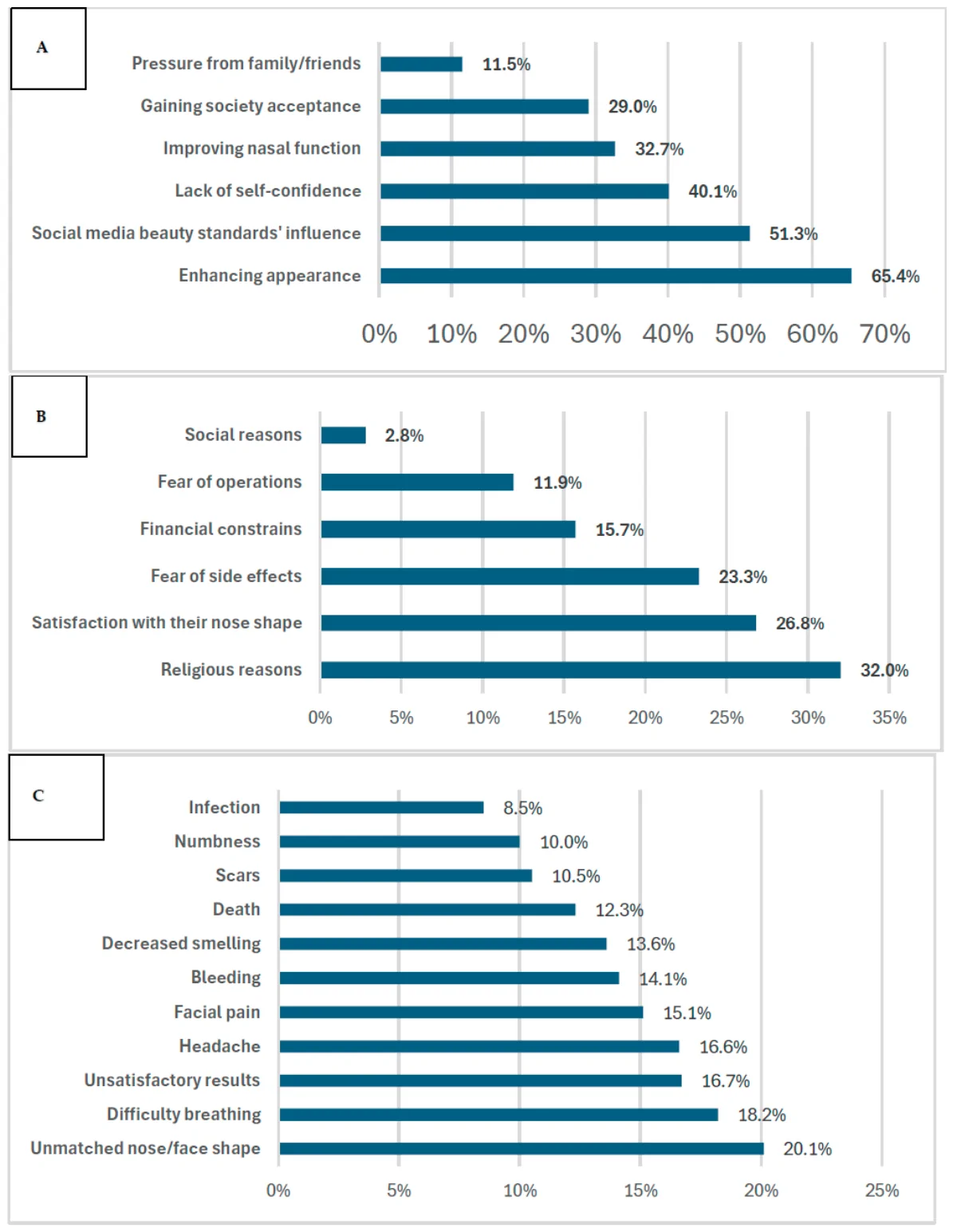
Participants’ perceptions about rhinoplasty. (**A**) Perceived reasons for undergoing rhinoplasty among study participants. (**B**) Perceived reasons against undergoing rhinoplasty among study participants. (**C**) Perceived seriousness of postoperative rhinoplasty complications.

The most common perceived reasons for undergoing rhinoplasty included enhancing appearance (65.4%), influence from social media beauty standards (51.3%), lack of self-confidence (40.1%), and improving nasal function (32.7%). Conversely, the most commonly cited reasons for not considering rhinoplasty were religious beliefs (32.0%), satisfaction with their nose shape (26.8%), and fear of side effects (23.3%). The most serious complications, as perceived by participants, were the mismatch between the nose and the face (20.1%), breathing difficulties (18.2%), unsatisfactory cosmetic outcomes (16.7%), headaches (16.6%), facial pain (15.1%), and bleeding (14.1%) (Figure 1A–C).

**Figure 2:**
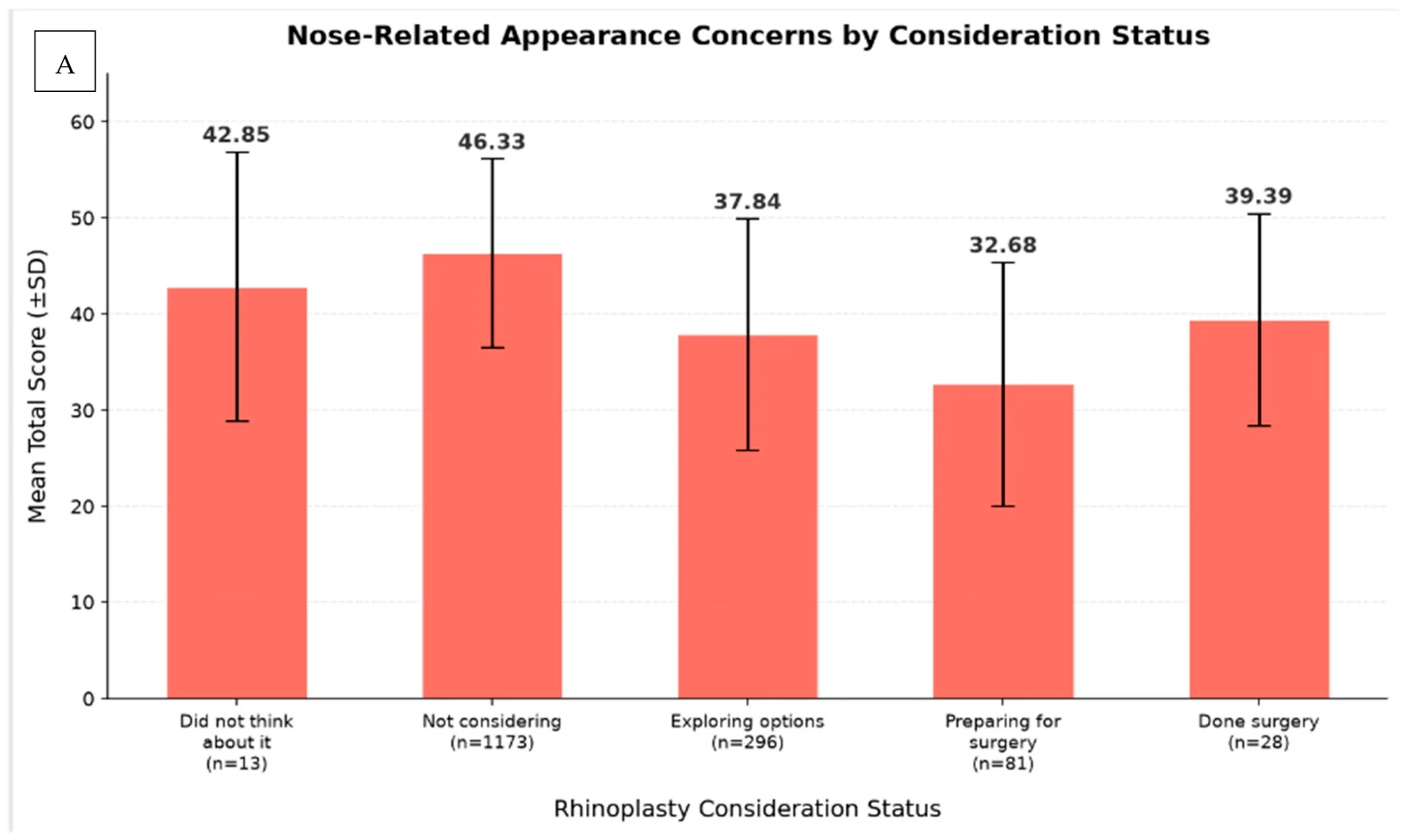

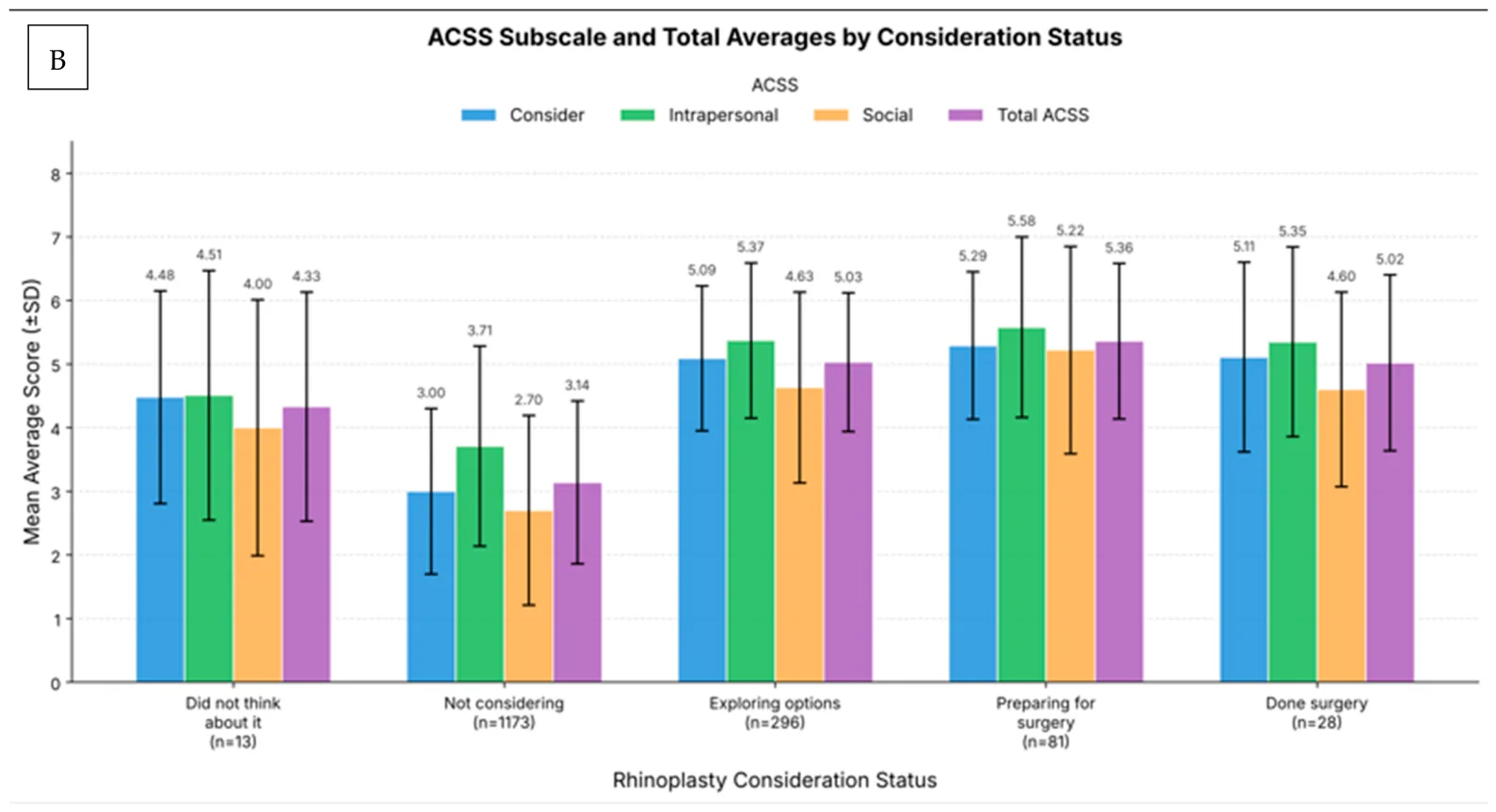
(**A**) Nose-related appearance concerns and (**B**) Acceptance of Cosmetic Surgery Scales among different groups.

Figure 2 shows variations in nose-related appearance concerns and ACSS scores across groups. Participants who were not contemplating rhinoplasty at all had significantly fewer concerns and lower acceptance scores for cosmetic surgery (ACSS) compared with other groups that are considering or have already undergone the surgery (*p*-value < 0.01), while no significant differences were detected between this group and the group that did not think about rhinoplasty.

A gradient in nose-related appearance concerns was observed, with higher scores indicating lower concerns. The scores decreased significantly from those not considering the surgery (46.3 ± 9.8) to those just exploring options (average 37.8 ± 12.0, *p* < 0.01), reaching the greatest concerns among those preparing for the surgery (32.7 ± 12.7, *p* < 0.01), followed by a rebound among those who have already undergone the surgery (39.4 ± 11.1, *p* < 0.01).

In the ACSS total score, participants preparing for surgery had significantly higher scores (5.4 ± 1.2) than those just exploring the options (5.0 ± 1.1, *p* = 0.04), and comparable scores to those who had undergone surgery (5.02 ± 1.2, *p* = 0.22).

Regression analysis was conducted to identify independent factors associated with considering or undergoing rhinoplasty. The ACSS was a significant independent factor associated with increased odds of considering rhinoplasty at all stages, from exploring options to preparing for and undergoing the surgery. At the stage of exploring options, economic status played a role in the decision; compared with participants with sufficient income, those with financial constraints had higher odds of exploring rhinoplasty options, while wealthier participants were 67% less likely to be at this stage (OR = 0.33, 95% CI: 0.16–0.71). For the more advanced stages (preparation and surgery), economic level was no longer significant, suggesting that finances are a factor in initial interest but not in follow-through once someone is committed. Greater nose-related concerns and having a peer who had undergone the surgery increased the odds that participants would explore or prepare for rhinoplasty. Older age was associated with increased odds of not thinking about rhinoplasty (OR = 1.11, 95% CI: 1.03–1.19) and decreased likelihood of exploring rhinoplasty options (OR = 0.98, 95% CI: 0.96–0.99). Being female was significantly associated with increased odds of undergoing rhinoplasty (OR = 7.76, 95% CI: 1.73–34.75). Table 3.

## 4. Discussion

This study investigated the factors associated with adults’ decisions to undergo surgery in Saudi Arabia. As individuals transition from “not considering” to exploring, to consultation, and ultimately to surgery, the associated motivating factors shift from economic constraints and nose-appearance concerns to peer influence and gender considerations. The acceptance of aesthetic surgery in general remains the sole consistent factor that increased the odds of considering rhinoplasty across all stages.

Participants in the current study perceived aesthetic and functional complications of rhinoplasty as more severe than less visible complications. Only a small proportion of participants considered the risk of death, infection, numbness, and other complications serious. Other studies from Saudi Arabia and other Arabic countries examined knowledge and awareness of rhinoplasty and its complications, confirming a substantially poor level of public awareness [7,18,19]. These findings highlight an important knowledge gap and underscore the need to improve awareness and preoperative counseling about the risks of elective cosmetic procedures to support informed decision-making, as these procedures require comprehensive risk awareness.

The present study demonstrated that the prevalence of considering rhinoplasty exceeds the prevalence of actually undergoing the procedure, a pattern consistently reported in recent regional studies [12,20,21] and attributable to financial, psychological, cultural, and safety-related concerns. Prior Saudi studies demonstrated a direct relationship between media exposure and increased desire to undergo rhinoplasty and other cosmetic procedures [22,23]. Our research aligns with this trend, with nearly three-quarters of participants citing social media as their primary source of rhinoplasty information and more than half perceiving social media beauty standards as a common reason for undergoing rhinoplasty. This high percentage underscores the significant influence of social media platforms in shaping community understanding and potentially driving interest in rhinoplasty procedures.

In the current study, being female was independently associated with increased odds of undergoing the surgery; this association was less evident in the early stages of exploring rhinoplasty options, consulting, or preparing for the surgery. Additionally, the prevalence of undergoing rhinoplasty was higher among female participants. These findings add to the available evidence, as many studies have reported higher acceptance of cosmetic surgery among females [24,25,26]. However, findings regarding rhinoplasty specifically remain inconsistent, with some studies demonstrating significant gender differences characterized by greater disagreement among males [20].

We reported a significant association between financial constraints and an increased likelihood of undergoing rhinoplasty. This association was evident in the early stages of considering rhinoplasty but not in the late stages of consulting and undergoing the surgery. These findings contradict a previous study that highlighted financial factors, such as the inability or unwillingness to afford aesthetic surgery, as barriers to patients seeking rhinoplasty primarily or exclusively for aesthetic reasons [27]. Additionally, a previous study reported that higher income levels were associated with greater motivation to pursue cosmetic surgery and a stronger influence of societal standards [28], suggesting that the relationship between financial status and acceptance of cosmetic procedures may vary across populations and underlying motivations. This effect of financial constraints on the odds of considering rhinoplasty should be considered a possible explanation rather than a causal interpretation, as the nature of the current study does not support the latter.

The current study found that the most common perceived reason for rejecting rhinoplasty was religious beliefs, consistent with previous research indicating that religious values can influence decisions regarding cosmetic surgery, with individuals holding stronger religious attitudes being less likely to pursue such procedures [20,29]. In Saudi Arabia, many elective cosmetic interventions may be viewed as religiously discouraged unless medically justified.

Both the current study and previous research demonstrate a significant association between having relatives or friends who have undergone rhinoplasty and an individual’s acceptance of the procedure, highlighting the important role of social influence in shaping personal decisions about cosmetic surgery [26,30].

In this study, greater nose-related appearance concerns were significantly associated with increased odds of considering rhinoplasty, consistent with previous research suggesting that lower self-ratings of attractiveness were associated with a greater likelihood of undergoing cosmetic surgery [31,32]. Interestingly, a previous study found a significant postoperative increase in self-esteem scores among individuals who underwent aesthetic rhinoplasty [9], suggesting the need for psychological evaluation in the pre- and postoperative management of patients considering rhinoplasty. It is worth noting that body image and self-enhancement were not directly measured in the current study, and their effects are considered a possible explanation warranting investigation in future research.

This study has several strengths: it addresses an increasingly relevant public health issue by examining factors associated with patients’ decisions to consider or undergo rhinoplasty. However, the cross-sectional design limits the ability to infer a causal relationship between the examined factors and the actual decision. Convenience sampling may limit the generalizability of the findings. We used the 11-item instrument to assess nose-appearance concerns; however, future research should conduct EFA and assess criterion/construct validity across diverse samples before the instrument can be considered fully validated. Further research should emphasize longitudinal designs and qualitative approaches, which may provide deeper insights into the psychological motivations and sociocultural factors underlying rhinoplasty acceptance and decision-making. It is important to develop interventions that promote informed decision-making and patient satisfaction in rhinoplasty procedures, including educational initiatives that enhance digital health literacy and media awareness, as well as public health campaigns that address body image, self-esteem, and the responsible use of social media.

## 5. Conclusions

More than one-quarter of the studied sample are willing to undergo or have already undergone rhinoplasty. Acceptance of cosmetic surgery in general was associated with increased odds of considering and undergoing rhinoplasty. Other factors, including having a friend or family member who has had the surgery and concerns about the appearance of the nose, may increase the likelihood of considering rhinoplasty. Many rhinoplasty complications are not recognized as serious drawbacks of the intervention.

## Figures and Tables

**Table 1:** Characteristics of study participants.

Characteristic N = 1591	N (%)
**Gender**	
Female	1113 (70%)
Male	478 (30%)
**Age group**	
Under 20 years	177 (11%)
20–40 years	819 (51%)
Over 40 years	595 (37%)
**Marital status**	
Married	949 (60%)
Unmarried	642 (40%)
Residence	
Central region	1123 (71%)
Northern region	59 (3.7%)
Southern region	135 (8.5%)
Western region	171 (11%)
Eastern region	103 (6.5%)
**Income level**	
Enough	1031 (65%)
Enough and saving money	173 (11%)
Not enough	238 (15%)
Not enough and in debt	149 (9.4%)
**Educational level**	
School graduate	543 (34%)
University education and above	1048 (66%)
**Source of information regarding rhinoplasty**	
Social media	1174 (74%)
Friends/family	472 (30%)
Rhinoplasty doctor	321 (20%)
Internet	663 (42%)
Others	23 (1.4%)
**Relatives/friends who had rhinoplasty**	
No	804 (51%)
Yes	787 (49%)
**Undergoing or contemplating rhinoplasty**	
Done surgery	28 (1.8%)
Preparing for surgery (had consultation/booked surgery)	81 (5.1%)
Just exploring options	296 (19%)
Not considering rhinoplasty at all	1173 (74%)
Did not think about it	13 (0.80)

Data are presented as frequency and percentages.

**Table 2:** Prevalence of considering and undergoing rhinoplasty among the studied sample.

Variable		Not Considering (*n* = 1173)		Just Exploring Options (*n* = 296)		Planning Surgery (*n* = 81)		Had Surgery (*n* = 28)		Did Not Think About It (*n* = 13)		*p*-Value
		N	%	N	%	N	%	N	%	N	%	
**Age (years)**	>40	464	78.0	84	14.1	29	4.9	7	1.2	11	1.8	<0.01 ^§^
	20–40	591	72.2	169	20.6	40	4.9	18	2.2	1	0.1	
	18–19	118	66.7	43	24.3	12	6.8	3	1.7	1	0.6	
**Gender**	Female	824	74.0	209	18.8	49	4.4	26	2.3	5	0.4	<0.01 ^§^
	Male	349	73.0	87	18.2	32	6.7	2	0.4	8	1.7	
**Marital status**	Unmarried	456	71.0	142	22.1	30	4.7	13	2.0	1	0.2	0.03 ^§^
	Married	717	75.6	154	16.2	51	5.4	15	1.6	12	1.3	
**Economic level**	Not enough and in debt	87	58.4	47	31.50	11	7.40	3	2.0	1	0.7	<0.01 ^#^
	Not enough	165	69.3	55	23.1	13	5.5	4	1.7	1	0.4	
	Enough	769	74.6	185	17.9	49	4.8	18	1.7	10	1.0	
	Enough and saving	152	87.9	9	5.2	8	4.6	3	1.7	1	0.6	
**Education**	School	388	71.5	118	21.7	28	5.2	5	0.9	4	0.7	0.08 ^§^
	University and higher	785	74.9	178	17.0	53	5.1	23	2.2	9	0.9	
**Relatives/friends who had rhinoplasty**	No	654	81.3	122	15.2	19	2.4	3	0.4	6	0.7	<0.01 ^§^
	Yes	519	65.9	174	22.1	62	7.9	25	3.2	7	0.9	

Data are presented as frequency and percentages. ^§^ Chi-square test used, ^#^ Fisher’s exact test used.

**Table 3:** Independent factors associated with considering and undergoing rhinoplasty.

**Had the surgery vs. not considering it at all**	
ACSS (modified)	2.42 (1.74–3.38)
Female	7.76 (1.73–34.75)
Friend/family member	6.92 (2.03–23.56)
**Preparing for surgery vs. not considering it at all**	
ACSS (modified)	2.48 (1.99–3.10)
Nose-related appearance concerns	0.95 (0.93–0.97)
Friend/family member	2.99 (1.69–5.29)
**Exploring the options vs. not considering it at all**	
ACSS (modified)	2.36 (2.07–2.69)
Nose-related appearance concerns	0.98 (0.97–0.99)
Age	0.98 (0.96–0.99)
**Economic factors (enough is the reference group)**	
In debt	2.47 (1.55–3.96)
Not enough money	1.58 (0.77–1.74)
Enough and saving	0.33 (0.16–0.71)
**Did not think about it vs. not considering it at all**	
Age	1.11 (1.03–1.19)

ACSS (modified): Acceptance of Cosmetic Surgery without Consider construct. Model fitting information: chi-square likelihood ratio test (x^2^ = 604.25, df 36, *p*-value < 0.01), Nagelkerke’s Pseudo R square = 0.31, goodness-of-fit deviance *p*-value = 1.000.

## Data Availability

Data will be available upon request from the corresponding author after approval from the IRB at PNU through their email irb@pnu.edu.sa.

## Supplementary Materials

The following supporting information can be downloaded at https://www.mdpi.com/article/10.3390/healthcare14203378/s1: File S1: Psychometric evaluation of nose-related appearance concern scale.
