# Supplementary material for: Factors Associated with Adults’ Decisions to Undergo Rhinoplasty in Saudi Arabia: A Cross-Sectional Study
Source: Healthcare (Basel). 2026 Oct 10;14(20):3378. doi: 10.3390/healthcare14203378 (PMC13704726; doi:10.3390/healthcare14203378)
Supplement: Supplementary file 1 [file healthcare-14-03378-s001.zip › healthcare-4448921-supplementary.pdf]

File S1: Psychometric evaluation of nose-related appearance concern scale

**Correlation Matrix**

| | | Avoid photography | Feel embarrassed | Feel inferior | Avoid leaving the house | Feel a freak | Distress when others stare | Adverse effect on marriage | Avoid school/ college /work | Feel isolated | Distress when not being able to go to social events | Distress when nose is seen in a mirror/window |
|---|---|---|---|---|---|---|---|---|---|---|---|---|
| **Correlation** | Avoid photography | 1.000 | .858 | .804 | .668 | .734 | .697 | .685 | .677 | .666 | .665 | .676 |
| | Feel embarrassed | .858 | 1.000 | .873 | .708 | .799 | .762 | .723 | .733 | .727 | .716 | .733 |
| | Feel inferior | .804 | .873 | 1.000 | .760 | .827 | .785 | .764 | .767 | .772 | .743 | .768 |
| | Avoid leaving the house | .668 | .708 | .760 | 1.000 | .830 | .680 | .827 | .857 | .849 | .819 | .821 |
| | Feel a freak | .734 | .799 | .827 | .830 | 1.000 | .781 | .809 | .822 | .821 | .801 | .813 |
| | Distress when others stare | .697 | .762 | .785 | .680 | .781 | 1.000 | .724 | .705 | .689 | .706 | .696 |
| | Adverse effect on marriage | .685 | .723 | .764 | .827 | .809 | .724 | 1.000 | .866 | .844 | .824 | .816 |
| | Avoid school/college/work | .677 | .733 | .767 | .857 | .822 | .705 | .866 | 1.000 | .909 | .861 | .872 |
| | Feel isolated | .666 | .727 | .772 | .849 | .821 | .689 | .844 | .909 | 1.000 | .879 | .874 |
| | Distress when not being able to go to social events | .665 | .716 | .743 | .819 | .801 | .706 | .824 | .861 | .879 | 1.000 | .860 |
| | Distress when nose is seen in a mirror/window | .676 | .733 | .768 | .821 | .813 | .696 | .816 | .872 | .874 | .860 | 1.000 |
| **Sig. (1-tailed)** | Avoid photography | | <.001 | <.001 | <.001 | <.001 | <.001 | <.001 | <.001 | <.001 | <.001 | <.001 |
| | Feel embarrassed | .000 | | .000 | .000 | .000 | .000 | .000 | .000 | .000 | .000 | .000 |
| | Feel inferior | .000 | .000 | | .000 | .000 | .000 | .000 | .000 | .000 | .000 | .000 |
| | Avoid leaving the house | .000 | .000 | .000 | | .000 | .000 | .000 | .000 | .000 | .000 | .000 |
| | Feel a freak | .000 | .000 | .000 | .000 | | .000 | .000 | .000 | .000 | .000 | .000 |
| | Distress when others stare | .000 | .000 | .000 | .000 | .000 | | .000 | .000 | .000 | .000 | .000 |
| | Adverse effect on marriage | .000 | .000 | .000 | .000 | .000 | .000 | | .000 | .000 | .000 | .000 |
| | Avoid school/college/work | .000 | .000 | .000 | .000 | .000 | .000 | .000 | | .000 | .000 | .000 |
| | Feel isolated | .000 | .000 | .000 | .000 | .000 | .000 | .000 | .000 | | .000 | .000 |
| | Distress when not being able to go to social events | .000 | .000 | .000 | .000 | .000 | .000 | .000 | .000 | .000 | | .000 |
| | Distress when nose is seen in a mirror/window | .000 | .000 | .000 | .000 | .000 | .000 | .000 | .000 | .000 | .000 | |

**KMO and Bartlett's Test**

| | | |
|---|---|---|
| Kaiser-Meyer-Olkin Measure of Sampling Adequacy. | | .963 |
| Bartlett's Test of Sphericity | Approx. Chi-Square | 23709.394 |
| | df | 55 |
| | Sig. | <.001 |

**Communalities**

| | Initial | Extraction |
|---|---|---|
| Avoid photography | .753 | .639 |
| Feel embarrassed | .842 | .740 |
| Feel inferior | .833 | .793 |
| Avoid leaving the house | .806 | .791 |
| Feel a freak | .823 | .836 |
| Distress when others stare | .694 | .659 |
| Adverse effect on marriage | .806 | .803 |
| Avoid school/college/work | .879 | .848 |
| Feel isolated | .878 | .839 |
| Distress when not being able to go to social events | .826 | .802 |
| Distress when nose is seen in a mirror/window | .829 | .815 |

Extraction Method: Principal Axis Factoring.

**Total Variance Explained**

| Factor | Initial Eigenvalues | | | Extraction Sums of Squared Loadings | | |
|---|---|---|---|---|---|---|
| | Total | % of Variance | Cumulative % | Total | % of Variance | Cumulative % |
| 1 | 8.783 | 79.841 | 79.841 | 8.567 | 77.878 | 77.878 |
| 2 | .720 | 6.544 | 86.385 | | | |
| 3 | .321 | 2.921 | 89.306 | | | |
| 4 | .216 | 1.966 | 91.271 | | | |
| 5 | .197 | 1.793 | 93.064 | | | |
| 6 | .166 | 1.506 | 94.571 | | | |
| 7 | .142 | 1.287 | 95.858 | | | |
| 8 | .137 | 1.246 | 97.104 | | | |
| 9 | .125 | 1.140 | 98.244 | | | |
| 10 | .109 | .992 | 99.236 | | | |
| 11 | .084 | .764 | 100.000 | | | |

Extraction Method: Principal Axis Factoring.

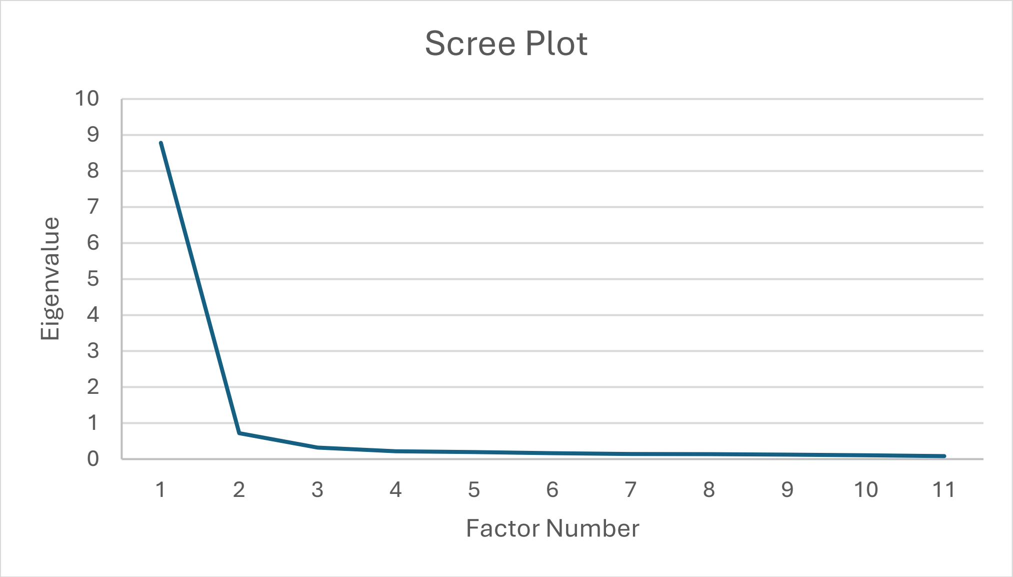


**Factor Matrix[a]**

| | Factor |
|---|---|
| | 1 |
| Avoid photography | .921 |
| Feel embarrassed | .916 |
| Feel inferior | .914 |
| Avoid leaving the house | .903 |
| Feel a freak | .896 |
| Distress when others stare | .896 |
| Adverse effect on marriage | .890 |
| Avoid school/college/work | .889 |
| Feel isolated | .860 |
| Distress when not being able to go to social events | .812 |
| Distress when nose is seen in a mirror/window | .800 |

Extraction Method: Principal Axis Factoring.

a. 1 factors extracted. 4 iterations required.

**Rotated Factor Matrix[a]**

a. Only one factor was extracted. The solution cannot be rotated.
